# Expression of oestrogen receptor-*β* in oestrogen receptor-*α* negative human breast tumours

**DOI:** 10.1038/sj.bjc.6603295

**Published:** 2006-08-01

**Authors:** G P Skliris, E Leygue, L Curtis-Snell, P H Watson, L C Murphy

**Affiliations:** 1 Department of Biochemistry & Medical Genetics, Manitoba Institute of Cell Biology, University of Manitoba, Winnipeg, Manitoba, Canada R3E OV9; 2 Department of Pathology, University of Manitoba, Winnipeg, Manitoba, Canada R3E OV9

**Keywords:** oestrogen receptor-*β* isoforms, breast cancer, immunohistochemistry, proliferation, basal phenotype

## Abstract

To analyse the phenotype of breast tumours that express oestrogen receptor-*β* (ER*β*) alone tissue microarrays were used to investigate if ER*β* isoforms are associated with specific prognostic markers and gene expression phenotypes in ER*α-*negative tumours. ER*α*-negative tumours were positive for ER*β*1 in 58% of cases (*n*=122/210), total ER*β* in 60% (*n*=115/192) and ER*β*2/cx in 57% of cases (*n*=114/199). Oestrogen receptor-*β*1 and total ER*β* were significantly correlated with Ki67 (*r*=0.28, *P*<0.0001, *n*=209; *r*=0.29, *P*<0.0001, *n*=191) and with CK5/6, a marker of the basal phenotype (*r*=0.20, *P*=0.0106, *n*=170; *r*=0.18, *P*=0.0223, *n*=158). ER*β*2/cx was strongly associated with p-c-Jun and NF-*κ*Bp65 (*r*=0.53, *P*<0.0001, *n*=93; *r*=0.35, *P*<0.0001, *n*=176). This study shows that a range of ER*β* isoform expression occurs in ER*α*-negative breast tumours. While expression of ER*β*1, total and ER*β*2/cx are correlated, individual forms show associations with certain phenotypes that suggest different roles in subsets of ER*α-*negative cancers. Based on our *in vivo* observations, ER*β* may have the potential to become a therapeutic target in the specific subcohort of ER*α*-negative breast cancers.

Oestrogen receptor-*α* (ER*α*) is an important biomarker of response to endocrine therapy in breast cancer (Osborne, 1998). However, the definition of ER status in breast cancer is potentially more complex, since there are now two known ERs, ER*α* and ER*β*. Oestrogen receptor-*β* is expressed in both normal and neoplastic human breast tissue (Leygue *et al*, 1998; Mann *et al*, 2001; Murphy *et al*, 2002; Fuqua *et al*, 2003; Skliris *et al*, 2003) but its role in either tissue remains unknown. Several isoforms of ER*β* have been identified, which are either exon deletions or products of alternative splicing which result in proteins that are truncated at the C-terminus and do not bind ligand (Lu *et al*, 1998; Ogawa *et al*, 1998; Fuqua *et al*, 1999; Leygue *et al*, 1999; Saunders *et al*, 2002). Thirty percent of breast tumours are classified as ER negative at the time of diagnosis and will be mostly resistant to endocrine therapy (Lapidus *et al*, 1998; Osborne, 1998). However, the previous assays used for ER measurement favoured the detection of ER*α* (Harvey *et al*, 1999; Brouillet *et al*, 2001) and we now know that some of these tumours express ER*β* (Murphy *et al*, 2003). Considering studies where ER*β* protein expression was determined, the pooled data sets were used to estimate the frequency of ER*β* and ER*α* status in breast cancers (Murphy *et al*, 2003). The most frequently occurring tumour type is ER*α*+/ER*β*+ (∼60%) with similar frequencies of the other three ER phenotypes (ER*α*+/ER*β*−; ER*α*−/ER*β*+; ER*α*−/ER*β*−) at 10–20% (Murphy *et al*, 2003). It is important to note that there are two groups of ER*β*-positive breast tumours, those with coexpression of ER*α* and those expressing ER*β* alone. The former is the most frequent and probably dominates the analysis of most previously reported correlative studies, and hence the positive association of ER*β* expression generally with good prognosis and good clinical outcome with respect to tamoxifen treatment (Mann *et al*, 2001; Omoto *et al*, 2001; Murphy *et al*, 2002; Iwase *et al*, 2003; Esslimani-Sahla *et al*, 2004; Fleming *et al*, 2004; Hopp *et al*, 2004; Myers *et al*, 2004; Nakopoulou *et al*, 2004). There is little data exploring tumours that express ER*β* alone. Under the current system of determining ER status, these are classified clinically as ER negative, and currently there are few markers for further subclassifying these ER*α*-negative cancers. Nevertheless recent data show that some invasive breast cancers expressing the basal cytokeratin CK5/6, may represent one ER*α*-negative subset, known as the basal epithelial phenotype and show a relatively poor prognosis (Perou *et al*, 2000; Sorlie *et al*, 2001, 2003; Nielsen *et al*, 2004). In the present study, we have investigated the level and frequency of expression of ER*β* in ER-negative tumours and its association with the basal phenotype and other established markers of prognosis, such as indicators of signal transduction pathways, proliferative and apoptotic markers.

## MATERIALS AND METHODS

### Tissues

All invasive breast cancers used in the current study were obtained from the Manitoba Breast Tumour Bank (MBTB, Department of Pathology, University of Manitoba) (Watson *et al*, 1996), which operates with approval from the Faculty of Medicine, University of Manitoba, Research Ethics Board. All samples included in the MBTB are rapidly frozen at −70°C immediately after surgical removal. A portion of the frozen tissue from each case is then processed to create matched formalin-fixed paraffin-embedded and frozen tissue blocks.

### Clinical–pathological characteristics of the patient cohort

Cases selected for this study were on the basis of (a) minimum patient follow-up of 36 months, (b) invasive components occupying more than 20% of the tumour section, while normal epithelial areas comprised no more than 10% of the epithelial content and (c) ER-negative status as defined by ligand binding analysis (LBA) of ⩽3 fmol mg^−1^ protein. The criteria for interpretation of the variables were as follows: (a) PR-positive status was defined as >15 fmol mg^−1^ protein by LBA; (b) grade, (Nottingham system), was assigned to low (scores 3–5), moderate (scores 6 and 7), or high (scores 8 and 9) categories; (c) tumour size, was assigned either small (⩽2 cm) or large (>2 cm) categories; (d) tumour inflammation was assessed by a scale from 1 to 5 and then assigned to low (scores 1–3) or high (scores 4 and 5) categories. All patients were treated with surgery and for 29 patients this was the only treatment regimen. The remaining patients received a variety of additional treatments, hormonal therapy (28), chemotherapy (49) or radiotherapy (9) alone, or combination of radiation followed by hormonal therapy (8), hormonal and chemotherapy (16), hormonal and chemotherapy (19) or chemotherapy (46), and for 6 patients the treatment regime was unknown.

### Tissue microarrays

The histopathology of all MBTB cases has been assessed and entered into a computerised database to enable selection based on composition of the tissue as well as clinical–pathological parameters. After selection, cases were rereviewed on H&E sections by a breast histopathologist (PHW). Tissue microarrays (TMAs) from a total cohort of 255 ER*α* negative (ER*α*–255TMA), primary invasive ductal breast carcinomas were constructed. Briefly, duplicate core tissue samples (0.6 mm diameter), were taken from selected areas of maximum cellularity for each tumour with a tissue arrayer instrument (Beecher Instruments, Silver Spring, MD, USA). Although the TMA consisted of 255 cases of ER-negative tumours as determined by LBA (ER+ >3 fmol mg^−1^ protein), 39 of these were subsequently found to be ER*α*+ by immunohistochemistry (IHC) and were excluded from the later analysis.

### Immunohistochemical assay

Serial sections (5 *μ*m) of the ER*α*–255TMA were cut, mounted on Fisherbrand Superfrost/plus slides (Fisher Scientific, USA) and stained using IHC with commercially available specific antibodies (Table 1). Further details of the three specific ER*β* antibodies are as follows: ER*β*1 (polyclonal, GC17/385P, Biogenex, CA, USA, raised to peptide containing amino acids 449–465) at 1 : 100 dilution; total ER*β* (monoclonal, 14C8, Genetex, TX, USA, raised to peptide containing amino acids 1–153) at 1 : 100; ER*β*2/cx (mouse monoclonal, clone 57/3, raised to synthetic peptide derived from the specific C-terminus of hER*β*2/cx isoform; Serotec, UK) used at 1 : 20. Briefly, sections were dewaxed in two xylene baths (5 min each), taken through a series of alcohols (100, 95, 70%), rehydrated in distilled water and then submitted to heat-induced antigen retrieval for 8 min in the presence of a citrate buffer (CC1 mild/standard, Ventana Medical Systems, AZ, USA) using an automated tissue immunostainer (Discovery Staining Module, Ventana Medical Systems, AZ, USA). The staining protocol was set to ‘Mild and Standard Cell Conditioning’ procedure for all antibodies. Primary antibodies were applied for 60 min (except for NF-*κ*Bp65 which were applied for 30 min) while secondary antibodies were incubated for 32 min. Initial dilutions quoted above were diluted further 1 : 3 with buffer dispensed onto the slide with the primary antibody. Primary antibodies were omitted for negative controls.

Total ER*β* IHC was performed manually; sections were microwaved in the presence of 0.01 M citrate buffer, pH 6.0, for 20 min at full power (Danby, ON, Canada, model DMW 1001 W, 800 W maximum output). Sections were blocked and then incubated using an ER*β* monoclonal antibody (14C8, Genetex, TX, USA) at 1 : 100 dilution in a humidified chamber at 4°C overnight, as previously described (Skliris *et al*, 2002, 2003; Fuqua *et al*, 2003). Following incubation with biotinylated goat anti-mouse antibody for 60 min at 1 : 200 (Jackson ImmunoResearch Laboratories, PA, USA) and with the Vectastain ABC kit (Vector Laboratories, CA, USA) for 45 min, total ER*β* protein was visualised with 3,3′-diaminobenzidine (DAB, Sigma-Aldrich, ON, Canada). Slides were scored semiquantitatively under a standard light microscope. Images were captured using Polaroid DMC-2 software (version 2.0.1, Polaroid, MA, USA).

### Quantification technique and marker selection

The expression of ER*β* isoforms (full-length-ligand binding ER*β*1, total ER*β* and ER*β*2/cx) and other prognostic markers was assessed using semiquantitative scoring (H-scores). H-scores derive from a semiquantitative assessment of both staining intensity (scale 0–3) and the percentage of positive cells (0–100%), which when multiplied, generates a score ranging from 0 to 300. Tissue microarray staining was evaluated by two authors (GPS, PHW) independently and where discordance was found, cases were re-evaluated together to reach agreement. For the primary categorical analysis, staining and cutoff points to distinguish low from high expression for each marker were as follows: only nuclear staining was evaluated for ER*β*1, total ER*β* and ER*β*2/cx isoforms and since there is no agreement or clinical relevant cutoff IHC-scores for ER*β* isoforms reported in the literature, several IHC-score cut-points equivalent to absent staining, the 25th percentile and median IHC-score values were tested in statistical analysis. Ki67, caspase-3 (markers of proliferation and apoptosis, respectively) and CK5/6 (a marker of the basal phenotype) were scored as previously described (Perou *et al*, 2000; Wykoff *et al*, 2001; Foulkes *et al*, 2004; El-Rehim *et al*, 2005). Since NF-*κ*B has been associated previously with more aggressive breast cancer (Biswas *et al*, 2004) and both NF-*κ*B and AP-1 have been shown to interact differentially with ER*α* and ER*β* (Paech *et al*, 1997; An *et al*, 1999) we have also assessed the relationship of ER*β* to these pathways in ER*α-*negative tumours. For NF-*κ*B/p65 nuclear staining was assessed and multiple H-score cutoffs were tested. P-c-Jun, a marker of AP-1 activity, was defined by nuclear staining and an H-score of >0.

### Statistical analysis

Associations between ER*β* isoforms and other clinical–pathological variables were tested using contingency methods (Fisher's exact test). Correlations were assessed by the Spearman's rank correlation test (*r*). Mann–Whitney rank sum tests, two-sided were also used to evaluate variables. Survival analyses were perfomed using the log rank test to generate Kaplan–Meier curves. Overall survival was defined as the time from initial surgery to the date of death attributable to breast cancer. Relapse-free survival was defined as the time from initial surgery to the date of clinically documented local or distant disease recurrence or death attributed to breast cancer. GraphPad Prism 4.02 version statistics software (GraphPad, San Diego, CA, USA) was used to perform all analyses.

## RESULTS

### Validation of ER*β* antibodies

**Figure 1:**
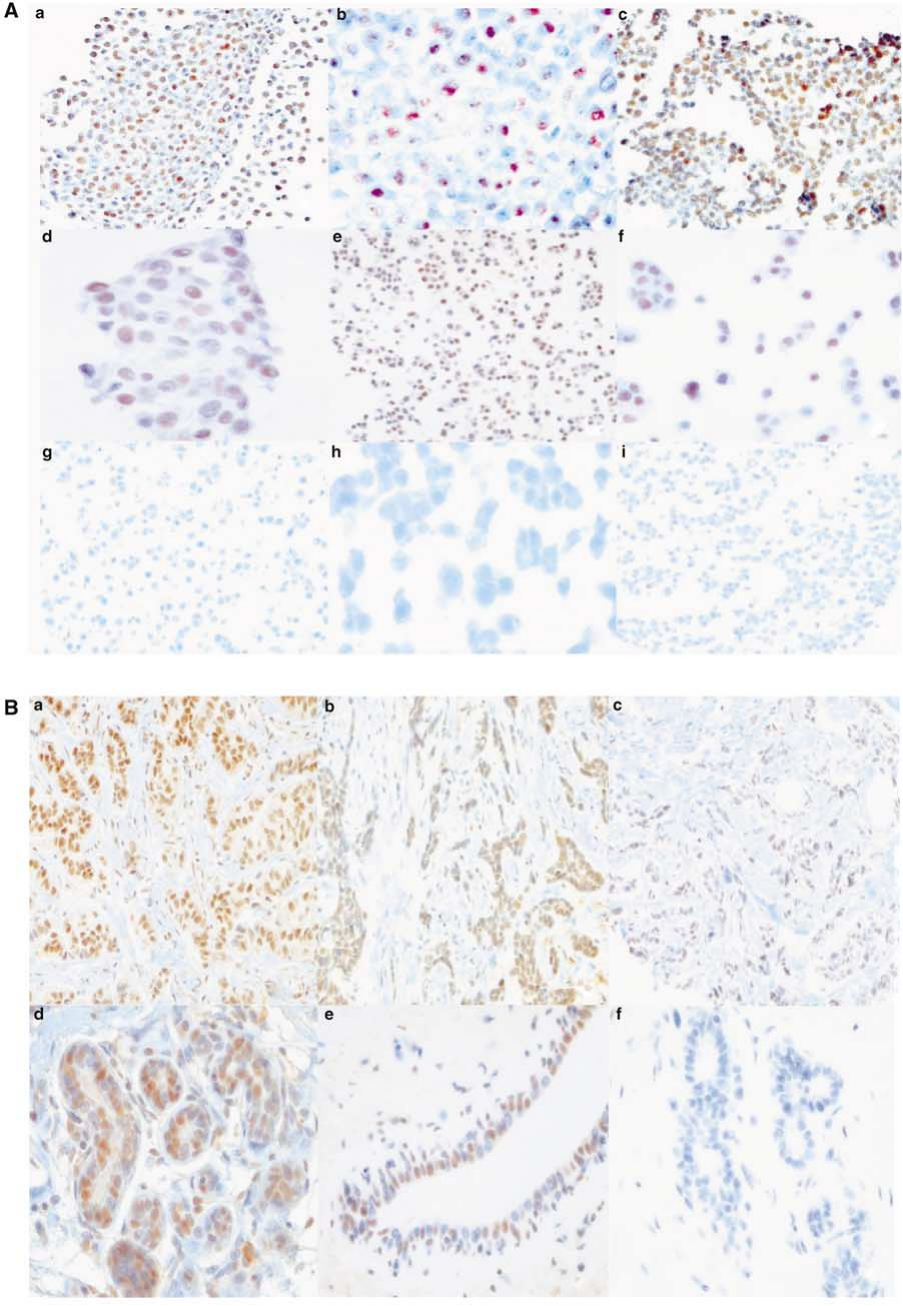
(**A**) Validation of ER*β*2/cx antibody (mouse monoclonal, clone 57/3, Serotec, UK): (a) Serotec clone 57/3 antibody staining of section from cell pellet of doxycycline treated tet-on-MDA231 cells stably overexpressing ER*β*2/cx, magnification × 500; (b) same as (a), magnification × 1250; (c) Serotec clone 57/3 antibody staining of section from cell pellet of doxycycline treated tet-on-MCF7 cells stably overexpressing ER*β*2/cx, magnification × 500; (d) same as (c), magnification × 1250; (e) Serotec clone 57/3 antibody staining of section from cell pellet of a separate clone of doxycycline treated tet-on-MCF7-cells stably overexpressing ER*β*2/cx, magnification × 500; (f) same as (e), magnification × 1250; (g) Serotec clone 57/3 antibody staining of section from cell pellet of doxycycline treated tet-on-MCF7 vector alone control cells, magnification × 500; (h) same as (g), magnification × 1250; (i) Serotec clone 57/3 antibody staining of section from cell pellet of doxycycline treated MCF7 stably overexpressing ER*β*1 (Murphy *et al*, 2005), magnification × 500. (**B**) Expression of ER*β*cx/2 in ER*α*-negative invasive tumours and normal breast tissue detected by IHC is demonstrated in representative panels. (a) Tumour core stained with the specific ER*β*cx/2 antibody (high H-score, 270); (b) tumour stained for ER*β*cx/2 (low H-score, 25); (c) tumour core showing negative staining for ER*β*cx/2 H-score, 0); (d) normal breast tissue showing strong, nuclear ER*β*cx/2 protein expression; (e) nuclear ER*β*cx/2 expression in normal breast ducts; (f) negative control (omission of ER*β*cx/2 antibody). Magnification × 500 for a, b, c, and × 1250 for d, e, f.

Three antibodies previously validated to detect ER*β* related proteins were used in this study (Fuqua *et al*, 1999; Leav *et al*, 2001; Saunders *et al*, 2002). GC17/385P (Leav *et al*, 2001) was raised to a C-terminal epitope of the wild-type ligand binding isoform of ER*β*, generally referred to as ER*β*1. 14C8 antibody (Fuqua *et al*, 1999) was raised to an N-terminal epitope which would be found in both ER*β*1 and multiple C-terminal truncated nonligand binding forms of ER*β* and therefore would detect multiple known ER*β* isoforms including ER*β*1 and ER*β*2cx. Hence we refer to it as detecting ‘total’ ER*β*. The antibody used to detect the nonligand isoform ER*β*2/cx (Saunders *et al*, 2002) has been previously validated by IHC and immunoblotting (Saunders *et al*, 2002). However, we have also validated the antibody further at the IHC level, by using MCF7 breast cancer cell lines, which have been engineered to overexpress ER*β*1 or ER*β*2/cx, after induction with the tetracycline analogue doxycycline (Murphy *et al*, 2005). Agar embedded cell pellets (Riera *et al*, 1999), formalin-fixed and paraffin-embedded (Adeyinka *et al*, 2002) from only the doxycycline treated cells expressing ER*β*2/cx but not ER*β*1 or controls were found to show nuclear staining with the specific ER*β*2/cx antibody under the same IHC conditions described above for the human breast tumours (Figure 1A).

### ER*β* isoform expression in ER*α-*negative human breast tumours

**Figure 2:**
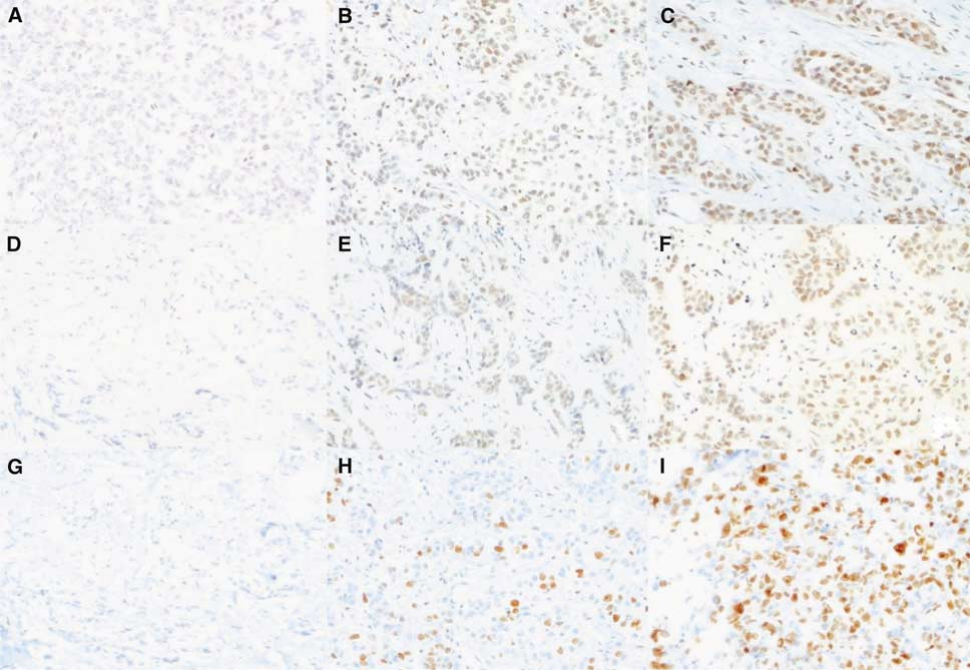
Expression of ER*β* and Ki67 in ER*α-*negative tissue microarray cores. (**A**–**C**) ER*α*-negative tumour cores stained with the specific ER*β*1 antibody (GC17/385P) showing negative, medium and high expression (a–c; H-scores of 0, 150 and 225, respectively); (**D**–**F**) ER*α*-negative tumour cores stained with total ER*β* antibody (14C8) showing negative, low and high expression (H-scores of 0, 25 and 100, respectively); (**G**–**I**) ER*α*-negative tumour cores showing negative, medium and high expression for Ki67, a proliferation marker (% positive, 0, 60 and 90%, respectively). Magnification × 500.

Serial sections of the ER*α*–255TMA were stained with specific antibodies for ER*β*1, total ER*β*, and ER*β*2/cx using IHC. Nuclear staining could be observed with ER*β*1 and total ER*β* antibodies in epithelial cells in our series of invasive cancers (Figure 2). Strong nuclear staining in both normal and neoplastic breast tissues for ER*β*2/cx isoform was often observed (Figure 1B). Using the 25% percentile of IHC-scores to define positive status for ER*β*1, total ER*β* and ER*β*2/cx, we observed that 58% of ER*α*-negative tumours were positive for ER*β*1 (*n*=122/210), 60% positive for total ER*β* (*n*=115/192) and 57% of cancers were positive for ER*β*2/cx (*n*=114/199; Table 2).

ER*β*1 was significantly correlated with both total ER*β* and ER*β*2/cx (*r*=0.28*, P*<0.0001, *n*=189; *r*=0.27, *P*=0.0002, *n*=196, respectively; Table 3). The same relationship was evident in categorical analysis using a variety of cutoff values for contingency analysis, where ER*β*1 was also significantly associated with ER*β*2/cx and total ER*β* (*P*=0.0083, >10; *P*=0.0016, 0.0391; >10, >25 respectively, Fishers exact test). Using a cut-point for ER*β*1 of either >10 or >25, median levels of total ER*β* expression were significantly higher in ER*β*1-positive *vs* -negative tumours (*P*=0.0026 and *P*=0.011, Mann–Whitney rank sum tests, two-sided). Similarly using the same two cut-points for ER*β*1 positivity median levels of ER*β*2/cx expression were significantly higher in ER*β*1-positive *vs* -negative tumours (*P*=0.0024 and *P*=0.022, respectively Mann–Whitney rank sum tests). These data suggest frequent coexpression of multiple ER*β* isoforms in breast tumours.

### Relationship of ER*β* isoform expression with markers of proliferation and apoptosis in ER*α*-negative human breast tumours

ER*β*1 (*r*=0.28, *P*<0.0001, *n*=209) and total ER*β* (*r*=0.29, *P*<0.0001, *n*=191; Table 3) were positively correlated with Ki67, a marker of proliferation, which was detected in the nuclei of ER*α-*negative tumours (Figure 2). Contingency analyses also showed that ER*β*1 and total ER*β* were associated with Ki67 (data not shown). Using the median Ki67 IHC-score as a cutoff to define low Ki67 (⩽25) and high Ki67 (>25), the median level of ER*β*1 expression was significantly lower in low Ki67 expressors (median ER*β*1=25) compared to high Ki67 expressors (median ER*β*1=50; *P*=0.0008, Mann–Whitney rank sum test). Similarly the median level of total ER*β* expression was significantly lower in low Ki67 expressors (median total ER*β*=20) compared to high Ki67 expressors (median total ER*β*=50; *P*=0.0008, Mann–Whitney rank sum test). No significant differences in ER*β*2/cx were found between the low and high Ki67 groups.

However, high proliferation in primary tumours prior to treatment, is often associated with high levels of apoptosis (Lipponen *et al*, 1994; Lipponen, 1999; Parton *et al*, 2002). Therefore, ER*β* expression was investigated with respect to a marker of apoptosis, active caspase-3 (Parton *et al*, 2002). No correlations were detected between ER*β* isoforms and caspase-3. However, Ki67 expression was significantly correlated (*r*=0.44, *P*<0.0001, *n*=211, Table 3) and associated (*P*<0.0001 Fisher's exact test; Mann–Whitney rank sum test) with caspase-3 in this breast tumour cohort. These data suggest that ER*β* expression in ER*α*-negative tumours is associated with markers of a high proliferative index.

### Relationship of ER*β* expression to basal epithelial phenotype markers in ER*α*-negative human breast tumours

Invasive breast cancers expressing the basal epithelial phenotype, based on the consensus of the published literature from cDNA microarray and IHC analyses, are ER*α* negative (Perou *et al*, 2000; Sorlie *et al*, 2001; Vijver *et al*, 2002; Nielsen *et al*, 2004; El-Rehim *et al*, 2005), CK5/6 positive (Sorlie *et al*, 2001; Korsching *et al*, 2002; Nielsen *et al*, 2004; Collett *et al*, 2005) and/or CK14 (El-Rehim *et al*, 2005) positive. The basal phenotype has also been associated with mutated BRCA1 (Foulkes *et al*, 2003, 2004; Sorlie *et al*, 2003; Collett *et al*, 2005). We were therefore interested to determine the relationship of ER*β* expression in ER*α*-negative tumours to markers of the basal epithelial phenotype. ER*β*1 and total ER*β* expression were weakly correlated with CK5/6 (*r*=0.20, *P*=0.010; *n*=170; *r*=0.18, *P*=0.022, *n*=158; Table 3). No correlations were seen with ER*β*2/cx. These data support the conclusion that many ER*α*-negative tumours expressing ER*β* are associated with some markers of a basal epithelial phenotype in breast cancer.

### ER*β*2/cx expression in ER*α-*negative human breast tumours

Despite the correlations and associations of ER*β*2/cx to ER*β*1 and total ER*β* described above, ER*β*2/cx was not correlated with Ki67 nor activated caspase-3. However, ER*β*2/cx was strongly correlated with p-c-Jun IHC-score (*r*=0.53, *P*<0.0001, *n*=93; Table 3). Contingency analyses for ER*β*2/cx and p-c-Jun positivity, identified a significant association of ER*β*2/cx with p-c-Jun (*P*<0.0001, Fisher's exact test). When p-c-Jun expression level was examined in relation to ER*β*2/cx status, p-c-jun IHC-score was significantly lower in ER*β*2/cx-negative tumours (median p-c-Jun=5) compared to high ER*β*2/cx expressors (median p-c-Jun=40; *P*<0.0001, Mann–Whitney rank sum test).

Similarly, ER*β*2/cx expression was also correlated with NF-*κ*Bp65 (*r*=0.35, *P*<0.0001, *n*=176; Table 3). Using either the 25% percentile (>0) or the median (>25) ER*β*2/cx IHC-score as cut-points to define negative and positive ER*β*2/cx status the median level of NF-*κ*Bp65 expression was significantly lower in negative/low ER*β*2/cx expressors (median NF-*κ*Bp65=50) compared to high ER*β*2/cx expressors (median NF-*κ*Bp65=100; *P*<0.0001, Mann–Whitney rank sum test). Similar but weaker relationships were found for total ER*β*. Using the median (>25) total ER*β* IHC-score as a cutoff to define negative and positive total ER*β* status the median level of NF-*κ*Bp65 expression was significantly lower in negative/low total ER*β* expressors (median NF-*κ*Bp65=75) compared to high total ER*β* expressors (median NF-*κ*Bp65=100; *P*<0.026, Mann–Whitney rank sum test). These data suggest that ER*β*2/cx expression is associated with AP1 and NF-*κ*B activity in ER*α-*negative breast tumours. A relationship between total ER*β* and p-c-Jun and NF-*κ*Bp65 was also demonstrated, and is likely to reflect the influence of the ER*β*2/cx component of the total ER*β* signal.

### ER*β* isoform expression in relation to clinical and pathological prognostic variables and survival

Only total ER*β* was associated with tumour grade (*P*=0.03). No other statistically significant associations between ER isoforms and established prognostic variables such as tumour size, age at diagnosis, node status, inflammation or progesterone receptor, were observed (Table 2, showing associations with cut-points equivalent to the 25th percentile).

**Figure 3:**
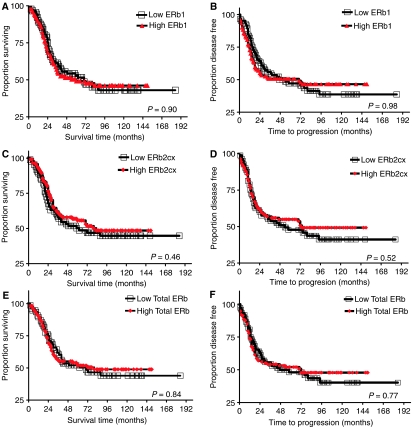
Kaplan–Meier graphs for ‘overall survival’ and ‘relapse-free survival-time to progression’ with respect to expression of ER*β*1 (**A** and **B**), ER*β*2cx (**C** and **D**) and total ER*β* isoforms (**E** and **F**, respectively). ER*β*1 overall survival (**A**), *n*=210, low ER*β*1 events=47, high ER*β*1 events=60. ER*β*1 time to progression (**B**), low ER*β*1 events=48, high ER*β*1 events=60. ER*β*2cx overall survival (**C**), *n*=199, low ER*β*2cx events=44, high ER*β*2cx events=53. ER*β*2cx time to progression (**D**), low ER*β*2cx events=44, high ER*β*2cx events=53. Total ER*β* overall survival (**E**), *n*=192, low total ER*β* events=40, high total ER*β* events=55. Total ER*β* time to progression, low total ER*β* events=40, high total ER*β* events=56.

Univariate survival analyses in relation to axillary nodal status, size, grade, Ki67, active caspase-3, or basal phenotype, showed a significant association only with nodal status (*P*=0.024) in this cohort of ER*α-*negative tumours. Furthermore no difference in disease outcome (overall survival and relapse-free survival) was found between low and high ER*β*1, total ER*β* or ER*β*2/cx (Figure 3).

## DISCUSSION

Several interesting observations have been made in the present study concerning ER*β* isoform expression in ER*α*-negative breast tumours. The first is that ER*β*1, total ER*β* and ER*β*2/cx isoforms are frequently expressed in this cohort of ER*α*-negative breast cancers. The second is that there is a significant correlation of ER*β*1 and total ER*β* with Ki67, a marker of proliferation, which is of particular interest. As this was not found when ER*β*2/cx expression was assessed, it is likely that the correlation with total ER*β* reflects the ER*β*1 component, although we cannot exclude the existence of other, as yet unknown variant isoforms. Indeed, the frequent expression of the ER*β* variant isoform, ER*β*5, in ER*α-*negative breast tumours has recently been described (Poola *et al*, 2005), however, we did not have access to specific antibodies to investigate this variant isoform in our breast tumour cohort. Our data confirm and extend an observation made by Jensen *et al* (2001), where the highest expression of either Ki67 and Cyclin A was found in tumours that only expressed ER*β*, indicating that ER*β* may be related to proliferation in breast cancer. Jensen's observation showing an association of ER*β* with Ki67, using an antibody that recognised total ER*β* (Jensen *et al*, 2001), also suggests that ER*β* isoforms are not only expressed in cells with the potential to cycle but also can be expressed in cells that are cycling. The existence of this relationship was reflected only in a very small subset of seven tumours in the ER*α*-negative/ER*β*-positive cohort in his study (Jensen *et al*, 2001), but a study by O'Neill *et al* (2004) published during the execution of our study confirmed his observation in a larger cohort (*n*=167). However, results from these latter studies came only from subset analysis of mixed cohorts of ER*α*-positive and -negative tumours. Our study is the only one so far exclusively focusing on ER*α*-negative cancers to address the issue of ER*β* expression. The cohort used in our study (*n*=216) is the largest so far and included tumours that were all selected to be ER*α* negative, both immunohistochemically and by LBA. Thus, the relationship of ER*β*1 alone expression in human breast cancer to Ki67, seems to be highly reproducible and therefore likely offers a new significant insight into the possible role of ER*β*1 in breast cancer. In contrast, this relationship is generally not seen in ER*α-*positive/ER*β*-positive breast tumours (Jarvinen *et al*, 2000; Mann *et al*, 2001; Omoto *et al*, 2001; Murphy *et al*, 2002; Fuqua *et al*, 2003; Iwase *et al*, 2003; Fleming *et al*, 2004; Hopp *et al*, 2004; Myers *et al*, 2004; Nakopoulou *et al*, 2004) and therefore our data together with two other studies support the conclusion that the role of ER*β*1 when expressed alone in human breast cancers *in vivo* is likely quite different to when it is coexpressed with ER*α*. Such data suggest that ER*β*1 may have a direct role in proliferation in ER*α-*negative breast cancers, but this is unproven.

The involvement of ER*β* isoforms in proliferation using cell line models is unclear. Most cell line models in which ER*β*1 has been stably expressed either inducibly or constitutively show that overexpression of ER*β*1 inhibits proliferation irrespective of whether it is coexpressed with ER*α* (Paruthiyil *et al*, 2004; Strom *et al*, 2004; Murphy *et al*, 2005) or not (Lazennec *et al*, 2001; Cheng *et al*, 2004). However, two studies using cell line models have been published in which stable constitutive overexpression of ER*β*1 resulted in increased proliferation (Tonetti *et al*, 2003; Hou *et al*, 2004) although in the former publication the short form of ER*β*1 (truncated by 45 amino acids from the N-terminus) was used. Both breast cancer cell lines used (MDA-MB-231 and MDA-MB-435) are typically ER*α* negative and therefore can be considered to represent the ER*β* alone expressing breast tumours cohort *in vivo*. However, in another constitutive ER*β* overexpression model based on the MDA-MB-231 cells, little or no effect on proliferation, positive or negative, was seen (Rousseau *et al*, 2004). Such data indicate that differences in potential cell line background, the type of ER*β* isoforms expressed and experimental variables including possibly clonal selection can influence the effect of ER*β* on proliferation. However, in other cancer cells types where ER*β*1 has been overexpressed, increased ER*β*1 is most often associated with inhibition of proliferation and/or increased apoptosis (Qiu *et al*, 2002; Cheng *et al*, 2004). It is unclear, however, whether the overexpression of ER*β*1 in experimental cancer cell line models, is relevant to the levels of ER*β*1 seen in tumours *in vivo*, especially since generally ER*β*1 expression is reduced in tumours compared to normal tissues in multiple cancers (Foley *et al*, 2000; Roger *et al*, 2001; Skliris *et al*, 2003) leading to the suggestion that ER*β*1 is a tumour-suppressor gene, and certainly would be consistent with the hypothesis that it is antiproliferative (Weihua *et al*, 2000; Forster *et al*, 2002; Paruthiyil *et al*, 2004). As well the possibility exists that ER*β*1 may be frequently mutated and/or altered post-translationally in breast cancers *in vivo*, although no published data as yet address this issue to our knowledge.

ER*β*1 and total ER*β* isoforms were also significantly correlated with CK5/6, a marker of the basal epithelial phenotype as defined from DNA microarray and IHC analyses, predominantly as ER*α* negative and CK5/6 positive (Sorlie *et al*, 2001; Korsching *et al*, 2002; Collett *et al*, 2005; El-Rehim *et al*, 2005). As ER*β* is found widely expressed in the basal myoepithelium (Murphy *et al*, 2002; Speirs *et al*, 2002) as well as in luminal epithelial cells in normal human breast tissues, it is possible that many ER-negative breast cancers expressing ER*β* are derived from a myoepithelial cell lineage, and that ER*β* is a marker of this lineage. Interestingly, a reduced myoepithelial cell layer is found in the lactating mammary gland of the ER*β* knockout mouse in contrast to the wild-type controls (Forster *et al*, 2002). This led to the hypothesis that ER*β* may be involved in regulating pathways, which are required for the differentiation of the myoepithelial cell lineage in the mammary gland (Forster *et al*, 2002).

While proliferation and the basal phenotype have been associated with poor survival, no differences in clinical outcome were identified between high and low Ki67 or any markers of the basal phenotype in our ER*α-*negative breast cancer cohort. It is possible that the lack of association of any of parameters investigated here (ER*β* isoforms, Ki67 and caspase-3) with clinical outcome (disease-free survival and overall survival) is confounded by the variety of treatments the patient cohort later received. In addition, most other studies where Ki67 has been examined as a prognostic factor have included both ER-positive and ER-negative tumours in their cohorts (Trihia *et al*, 2003). It should also be noted that ER*α-*negative status in our cohort was defined by negative IHC and ligand binding assay. This definition eliminated 15% of an initial ER*α-*negative series selected only on the basis of ligand binding assay. A similar number of ER*α* IHC-negative tumours have been found to be positive by ligand binding assay (Huang *et al*, 1997). The basis for discrepancy between these two ER*α* assays has been a subject of past discussion in the literature (Huang *et al*, 1997), but is likely to reflect biological variables rather than tissue selection or composition, because of the design of our tumour bank. Therefore, the current study used stringently defined ER*α*-negative tumours and so was enriched for a generally more aggressive group of breast tumours.

In comparison to ER*β*1, the role of its variant, ER*β*2/cx, is even more unclear. Transient expression studies using human ER*β*2/cx, have shown that human ER*β*2/cx is unable to bind ligand and when overexpressed sufficiently can inhibit ER*α* transcriptional activity (Ogawa *et al*, 1998; Peng *et al*, 2003) but has little if any effect on ER*β*1 activity. In breast cancer ER*β*2/cx has been identified at both the RNA and protein levels (Saji *et al*, 2002; Esslimani-Sahla *et al*, 2004), and now with another antibody we have also shown the presence of ER*β*2/cx in both normal and neoplastic breast tissue. Most studies previously published suggested that ER*β*2/cx is increased in breast tumours compared to normal breast tissue (Omoto *et al*, 2002; Palmieri *et al*, 2004) and the relative expression of the ER*β*2/cx to ER*β*1 is likely to change during breast tumourigenesis. However, no studies focusing only on ER*α*-negative tumours have been published. A few studies have suggested hypotheses as to ER*β*2/cx function due to observed correlations and association with other prognostic markers and clinical outcome with or without treatment (Omoto *et al*, 2002; Esslimani-Sahla *et al*, 2004; Palmieri *et al*, 2004). Esslimani-Sahla *et al* (2004) showed that ER*β*2/cx expression was correlated with total ER*β*, which is in agreement with our observation in our ER*α-*negative series. However, among these studies contradictory conclusions have often been reached (Saji *et al*, 2002; Esslimani-Sahla *et al*, 2004; Palmieri *et al*, 2004). Our data suggest that in ER*α-*negative tumours, ER*β*2/cx expression is significantly associated with both increased AP-1 and NF-*κ*B expression and that ER*β*1 may not be associated with these activities. This suggests that the different ER*β* isoforms may be involved in regulation of distinct pathways in these tumours or alternatively there is differential regulation of ER*β* isoforms by distinct pathways in these tumours.

The absence of any significant correlations between ER*β* isoforms and particularly total ER*β* with either overall or relapse-free survival is also in agreement with some other published studies (Hopp *et al*, 2004) but disagrees with other studies where increased ER*β* has been associated with better survival (Nakopoulou *et al*, 2004) and when patients were treated with tamoxifen alone, where an association was shown with better response to tamoxifen therapy (Murphy and Watson, 2006). However, in these latter studies the majority if not all the tumours assessed were ER*α* positive and so represent a different context where ER*β* is coexpressed with ER*α*. In the current study we have hypothesised that the function of ER*β* expressed alone will be different to that when ER*β* is coexpressed with ER*α*, and therefore we have looked at a distinct cohort of patients where their tumours are ER*α* negative.

These data support the hypothesis that the role of ER*β* expression is different when expressed alone, to its role when coexpressed with ER*α* in human breast cancer. This is specifically reflected in the present study, by the confirmation of a strong relationship of ER*β*1 with Ki67 in ER*α-*negative tumours, such that it seems likely that the addition of an ER*β*1 antagonist could be a potentially useful therapy in specific subsets of breast cancer patients in a clinical setting.

## Figures and Tables

**Table 1:** Details of antibodies used for IHC in the present study

**Antibody**	**Antibody clone**	**Supplier**	**Antibody dilution**	**Incubation details**	**Antigen retrieval method^a^**
ER*β*	385P polyclonal	Biogenex, USA	1 : 100	1 h @ 42°C	CC1
Total ER*β*	14C8	Genetex, USA	1 : 100	O/N @ 4°C	0.01 M citrate pH 6.0^b^
ER*β*2/cx	57/3	Serotec, UK	1 : 20	1 h @ 42°C	CC1
CK5/6	D5/16134	Zymed Labs, USA	1 : 20	1 h @ 42°C	CC1
Her2/neu	CB11	Novocastra, UK	1 : 50	1 h @ 42°C	CC1
EGFR	3C6	Ventana Systems, USA	Dispensed	30 min @ 42°C	Protease 1^c^
Ki67	MIB-1	Dako, Canada	1 : 50	1 h @ 42°C	CC1
Caspase-3	Asp175	Cell Signaling, USA	1 : 100	1 h @ 42°C	CC1
p-c-Jun	822	SantaCruz, USA	1 : 100	1 h @ 42°C	CC1
NF-*κ*Bp65	8008	SantaCruz, USA	1 : 625	30 min @ 42°C	CC1
ERα	6F11	Novocastra, UK	1 : 50	1 h @ 42°C	CC1

ERα, oestrogen receptor-*β;* IHC, immunohistochemistry.

a Mild and standard cell conditioning, using CC1 antigen retrieval buffer (Ventana Medical Systems, AZ, USA).

b IHC procedure performed manually.

c Ventana Automated Systems using protease-1 enzyme for antigen retrieval.

**Table 2:** Clinical and pathological characteristics of the study cohort

	**ER*β*1 IHC-score^a^**			**Total ER*β* IHC-score^a^**			**ER*β*2/cx IHC-score^a^**		
**Patient characteristics**	**Number (*n*)**	**%**	***P*-value**	**Number (*n*)**	**%**	***P*-value**	**Number (*n*)**	**%**	***P*-value**
*ERα status*									
−ve	210	100		192	100		199	100	
									
*PR status*									
−ve	183	87	0.89	167	87	0.99	175	88	0.15
+ve	27	13		25	13		24	12	
									
*ERβ1 status*									
+ve	122	58							
−ve	88	42							
									
*Total ERβ status*									
+ve				115	60				
−ve				77	40				
									
*ERβ2/cx status*									
+ve							114	57	
−ve							85	43	
									
*Grade*									
Low	24	11		22	12		22	11	
Mod	75	36	0.28	66	34	**0.03**	69	35	0.10
High	111	53		104	54		108	54	
									
*Tumour size (cm)*									
⩽2	58	28	0.68	55	29	0.50	55	28	0.42
>2	152	72		137	71		144	72	
									
*Inflammation*									
Low	130	64	0.46	118	62	1.0	120	62	0.09
High	74	36		71	38		73	38	
									
*Age (years)*									
⩽50	71	34	0.82	65	34	0.54	66	33	0.71
>50	139	66		127	66		133	67	
									
*Node status*									
0	100	48	0.67	92	48	0.57	94	47	0.08
1	110	52		100	52		105	53	
									
*Metastasis*									
Distant	72	73	0.76	64	74	0.72	65	73	0.61
Regional	18	18		15	17		15	17	
Local	9	9		7	9		9	10	

ERα, oestrogen receptor-*β;* IHC, immunohistochemistry.

a =Using the 25th percentile of IHC-scores to define positive status for ER*β*1, total ER*β* and ER*β*2/cx (>10, >10, >0). *P*-value was obtained by using Fisher's exact test.

Values in bold are statistically significant (*P*<0.05).

**Table 3:** Spearman rank correlations of ER*β* isoforms with other prognostic markers

	***r* (Spearman)**	***P*-value**	**No. of cases (*n*)**	**Correlation with**
ER*β*1	0.27	0.0002	196	ER*β*2/cx
	0.28	<0.0001	189	Total ER*β*
	0.28	<0.0001	209	Ki67
	0.20	0.010	170	CK5/6
				
ER*β*2/cx	0.44	<0.0001	178	Total ER*β*
	0.53	<0.0001	93	p-c-Jun
	0.35	<0.0001	176	NF-*k*Bp65
				
Total ER*β*	0.29	<0.0001	191	Ki67
	0.18	0.022	158	CK5/6
	0.24	0.020	88	p-c-Jun
	0.24	0.002	169	NF-*k*Bp65
				
CK5/6	0.20	0.006	171	Ki67
	0.19	0.014	159	NF-*k*Bp65
	0.23	0.006	133	EGFR
				
NF-*k*Bp65	0.32	<0.0001	159	Her-2/neu
				
Caspase-3	0.44	<0.0001	211	Ki67
	0.18	0.021	156	EGFR

ERα, oestrogen receptor-*β;* IHC, immunohistochemistry.
